# A Feasibility Study of a Non‐Professional‐Led Multi‐Component Psychosocial Support Program Incorporating Laughter and Music for People Living with Dementia and Their Families

**DOI:** 10.1002/alz70858_104279

**Published:** 2025-12-26

**Authors:** Aya Seike, Sayaka Takeuchi, Junko Hagihara, Chiharu Moriyama, Shigemi Nanpo, Keiko Hara, Rieko Inokuchi, Yumi Shigesada, Tomohiko Takahashi, Shigenori Tanaka, Hideya Shoji, Yumeka Kobayashi, Rin Mizuno, Kodai Kawase, Yuichiro Yano, Akinori Takeda, Takashi Sakurai, Hidenori Arai

**Affiliations:** ^1^ Faculty of Sports and Health Science, Ritsumeikan University, Kusatsu city, Shiga, Japan; ^2^ National Center for Geriatrics and Gerontology The Center for Comprehensive Care and Research on Memory Disorders, Ohbu, Japan; ^3^ Division of Regional Development System Design, Graduate School of Agriculture, Hokkaido University, Sapporo, Hokkaido, Japan; ^4^ National Center for Geriatrics and Gerontology The Center for Comprehensive Care and Research on Memory Disorders, Ohbu, Aichi, Japan; ^5^ National Center for Geriatrics and Gerontology, The Center for Comprehensive Care and Research on Memory Disorders, Ohbu, Aichi, Japan; ^6^ Faculty of Sports and Health Science, Ritsumeikan University, Kusatsu, Shiga, Japan; ^7^ Department of General Medicine, Juntendo University Faculty of Medicine, Tokyo, Tokyo, Japan; ^8^ Juntendo University AI Incubation Center, Tokyo, Japan; ^9^ National Center for Geriatrics and Gerontology, Obu, Aichi, Japan

## Abstract

**Background:**

Globally, over 55 million people are living with dementia, with more than 10 million new cases annually (WHO, 2023), creating an urgent need for interventions to maintain the quality of life (QOL) of both people living with dementia (PLWD) and their family carers (hereafter “families”). In Japan, initiatives like dementia cafés emphasize simultaneous support for PLWD and families, but evidence of their effectiveness remains limited. This study evaluated the feasibility of a group‐based, multi‐component psychosocial support program incorporating entertainment elements such as comedy and music, facilitated by non‐professionals to reduce participation barriers.

**Method:**

The study employed a single‐arm intervention trial over 12 weeks. Eligible PLWD were aged 65–90 years, diagnosed with mild cognitive impairment or mild to moderate dementia (MMSE ≥15), and living at home. Families included carers aged 20–90 years, regardless of cohabitation, who attended all sessions with PLWD. The program featured four key elements: simultaneous participation of PLWD and families, a group‐based format, a needs‐based multi‐component approach (reminiscence therapy, stress management, and laughter‐focused recreational activities), and non‐professional facilitation. The program included six sessions (three music‐based and three comedy‐based) over three months, accommodating 10 participants (5 pairs) per cycle.

**Result:**

Ten PLWD and 10 families completed the intervention. Among PLWD, Alzheimer's disease was the most common diagnosis, with a disease duration of 2.5–3 years. Over 70% required assistance at Support Level 2 or Care Level 1, with an average caring duration of 4 years. The dropout rate was below 25%, and participant satisfaction averaged 84.5/100 points, indicating high acceptance. For PLWD, cognitive and daily living functions showed improvements (MMSE: Δ 2.4; Barthel Index: Δ 2.9). For families, depressive symptoms improved slightly (CES‐D: Δ ‐0.5), though caring burden (J‐ZBI: Δ 3.6) did not improve.

**Conclusion:**

The program demonstrated feasibility and preliminary effectiveness in improving outcomes for PLWD and families. High participant satisfaction highlights its potential as a scalable psychosocial intervention model. A randomized controlled trial involving 120 pairs is underway to further evaluate its impact on dementia care practices and family support systems.

